# Ubuntu considered in light of exclusion of people with disabilities

**DOI:** 10.4102/ajod.v7i0.460

**Published:** 2018-11-29

**Authors:** Sindile A. Ngubane-Mokiwa

**Affiliations:** 1 College of Graduate Studies, University of South Africa, South Africa

## Abstract

**Background:**

This article emanates from a study funded by the KwaZulu-Natal chapter of South Africa’s National Research Foundation on the ‘Archaeology of Ubuntu’. It explores the notion of ubuntu and disability in a group of Zulu people from four communities within KwaZulu-Natal. The study is based on the notion that ubuntu is humaneness. Being human is linked to notions of care, respect and compassion.

**Objectives:**

The article explores the treatment of people with disabilities from the elders’ perspectives in this community.

**Method:**

This article is based on qualitative data resulting from structured interviews conducted in the KwaZulu-Natal Province between February and March 2015.

**Results:**

The results reveal that society considered the birth of a disabled child as a curse from God and punishment from the ancestors. The results also indicate that people with disabilities were excluded from community activities; marrying a disabled person was unthinkable because they were stigmatised and dehumanised. The work of Hannah Arendt is used to interrogate people’s perceptions of others with disabilities in their communities.

**Conclusion:**

The article posits that treatment of people with disabilities is not cast in stone but can be renegotiated and restructured through community engagement to represent genuine inclusion.

## Introduction and background

*Ubuntu* is an African view that grounds societies that embrace communal ways of living. This means that one is not considered a human being unless one is concerned about the well-being of other people. One of the main characteristics of ubuntu is communality or communal well-being. This principle is based on the Nguni saying: *Umuntu ngumuntu ngabantu* [A person is a person through other people]. Letseka (2012) postulates that ubuntu is based on the etiquette of humanity, which includes caring for each other’s well-being and reciprocating kindness. Louw (2003) sees ubuntu as more than just being who you are through others; he extends ubuntu to how people relate to others around them. Louw’s concept of ubuntu emphasises the importance of having a mutual understanding of how people treat each other. Different authors have defined *ubuntu* as the ethic of care (Waghid & Smeyers 2012) and ‘a theory of right action’ or ‘moral theory’ (Letseka 2000; Metz 2007; Teffo 1994), as a pedagogical principle (Letseka 2013) and as a constitutional and jurisprudence principle (Mahao 2010; Mokgoro 1998). As ubuntu concerns the way in which people treat each other, this article focuses mainly on the treatment of people with disabilities living in the Zulu cultural communities. The main research objective guiding the study was to establish how such people were treated. The research also sought to investigate how the traditional elders within these communities understood and practised ubuntu with regard to people with disabilities.

This article is divided into five sections. In the first section, I introduce the philosophy of ubuntu and how people with disabilities have been treated in the selected communities. In the second section, I describe the research methodology that guided this qualitative research study, which was conducted through oral histories and structured interviews. This section also presents the manner in which ethical considerations, sampling techniques and data analysis were tackled. In the third section, I present findings, using verbatim quotes from the research participants with the aim of indicating the link between ubuntu and disability. In the fourth section, I discuss the research findings, making some recommendations for how people with disabilities should be better included and treated, according to ubuntu principles. Lastly, a conclusion is drawn.

The exclusion of children with disabilities is not a practice limited to African society: Peter Singer, a bioethicist, argued that the trouble of disability for the child and the parents further outweighed emotional choices, judgements and the communities’ socio-economic realities (Singer 1992). Singer’s utilitarian argument did not go unchallenged as it was based on a negative perception of people with disabilities. Harriet McBryde Johnson, an activist with a disability, questions whether her existence with a disability causes her to live a miserable life. She asserts that people with disabilities have the same feelings as other people and also have their own unique ways of doing things, her main argument being that ‘the presence or absence of disability doesn’t predict quality of life’ (Johnson 2003).

## Ubuntu as inclusion

In forming a basis for the position of this article, I first define the parameters within which the concept of inclusion is discussed. These include access, participation and school governance, curriculum and identity (Sayed et al. 2007). In this context, *access* would mean that people with disabilities participate meaningfully in all community activities. That would include the birth celebrations, initiation ceremonies, weddings, all forms of traditional dances, choosing marriage partners, becoming parents and grandparents. Participation in governance by such people would mean that they too can contribute towards lawmaking processes and that they can contest and be elected to leadership positions as well as have their views respected in the community. *Curriculum*, in the case of the community, refers to the different life stages that each child is initiated into and taught about through oral tradition and teachings hidden in idioms and proverbs. In the case of the AmaZulu, a child with a physical impairment would have to be afforded the opportunity to go through initiation. Because identity formation is a crucial element of any living human being, communities with ubuntu values would have to find ways of ensuring that their identity perceptions do not make those who differ from the norm feel devalued.

In discussing inclusion, one cannot avoid discussing what would amount to exclusion in a communal context. According to Sen (2000), *exclusion* concerns the way people with disabilities experience being among other people, how the former are excluded from participation in pedagogical, economic, social and political activities and are subjected to systemically engineered exclusion from equal development opportunities.

## Ubuntu among the Zulu culture through Hannah Arendt’s lens

The views of community elders on how people with disabilities were treated, as collated from the KwaZulu-Natal areas of Umgungundlovu, eThekwini, kwaHlabisa and uMtubatuba, are presented in this article. The cohort of elders comprised three males and six females aged between 65 and 97. To understand the practices as expressed by the elders, I use Hannah Arendt’s (1958) lens of critical and political theory, with reference to humanness. Arendt (1958:32–33) argues that societies are made of distinctive human beings who form an organised entity. She postulates that in this structure there are powerful community members who determine the rules and regulations of how people should behave. In this regard, the lawmakers need to be clear on how the community should behave towards the vulnerable members of the community, including children, women and people with disabilities. Arendt suggests that community rules should be made with the realisation that human frailty is a universal phenomenon (Siebers 2007). This means that disability is not confined to certain people; anybody could find themselves having to deal with it in their family or at a certain stage of their life. Arendt makes a call to ensure that all community norms are based on the right to full political participation. This plea resonates with the principle of ubuntu as identified by Sayed et al. (2007), who assert that people with disabilities should also participate in governing societal practices.

Arendt’s existentialism is considered adequate as a lens to analyse the ubuntu philosophy among the Zulu culture. For Arendt’s existentialism to be able to operate, the societal system should recognise people with disabilities as equal beings within the community. For this to happen there should be differences in the way humanness is identified. Humanness would need to transcend ability and similarity in bodily features. Humanness would also need to accept multidiversity in terms of physical traits (skin complexion, height, weight, presence or non-presence of limbs, functional or non-functional limbs, etc.), sensory capacities (hearing, vision, etc.) and so on. Humanness, which the elders equated to ubuntu, should be about acceptance of another human being in all shapes and forms, affording people with disabilities the same care, dignity and teachings that will make Zulu society into *abantu abaqotho* [principled people with ubuntu values].

## Research methodology

This article is based on qualitative data resulting from structured interviews conducted in the KwaZulu-Natal Province between February and March 2015. The sample of three elderly men and six elderly women between the ages of 65 and 100 who participated in the study was drawn through purposive sampling. While this article is based on data obtained from the said province, in particular, the wider study covered Southern African countries such as Botswana, Lesotho, Namibia, Swaziland, Zambia and Zimbabwe. It also covered four provinces in South Africa: KwaZulu-Natal, Limpopo, Mpumalanga and North-West. The qualitative study is based on oral historical conversations, mainly structured interviews with a group of Zulu people from the four communities mentioned within KwaZulu-Natal.

According to Jewsiewicki and Mudimbe (1993), ‘oral historiographies from a sense of the past creates a link between the past, the present, and the future of Africa’. They further state, ‘oral historiographies have always been bearers of norms and of logical systems for the interpretation of the past’. Vansina (1985) points out the strength of using oral historiographies when documenting historical events from traditional societies. Traditional societies are known to be resourceful in narrating historic accounts, hence creating a link between ‘the past, the present and the future of Africa’ (Jewsiewicki & Mudimbe 1993). Vansina (2006) argues that those who can read and write nevertheless regard oral historiography as a valuable research method.

Initially, the researchers had planned to engage 20 elders, comprising 10 males and 10 females. However, ultimately they only engaged with nine elders consisting of six females and three males. Some of the elders originally identified were ill and could not articulate themselves clearly, while a few of the others were not available. Table 1 illustrates the elders’ pseudonyms, their real gender and age.

**TABLE 1:** Profile of the research participants (community elders by gender and age).

Participants	Gender	Age (years)
Gogo A	Female	85
Gogo B	Female	79
Gogo C	Female	95
Gogo D	Female	67
Gogo E	Female	69
Gogo F	Female	90
Mkhulu A	Male	98
Mkhulu B	Male	77
Mkhulu C	Male	69

Note: *Gogo* is a respectful title given to an elderly woman; the direct meaning is ‘grandmother’. *Mkhulu* is a respectful title given to an elderly man; the direct meaning is ‘grandfather’.

### Ethical considerations

This research study on the ‘Archaeology of Ubuntu’ was conducted after obtaining consent from the university. The committee is guided by the research ethics guidelines laid down by the university. The policy calls for researchers to be ‘competent and accountable’, to have ‘integrity’, to conduct research that seeks to benefit society, develop ‘knowledge creation’, to prevent ‘harmful consequences’ and ‘misuse and misrepresentation’ of their research work. It further assigns to the principal researchers the responsibility of ensuring ‘ethical conduct’ by research juniors. This research policy also binds researchers to afford the research participants their rights, sovereignty and right to benefit from research (Seidman 2013; Unisa 2013).

The research participants voluntarily participated in the study with the guarantee that their identity would not be divulged as per the ethical requirements. Pseudonyms are used to safeguard the real identity of the participants. To avoid any issues such as ‘everyday memory problems’ (Vemuri 2004:202) the interviews were audio recorded. The recordings were transcribed and translated from isiZulu to English by a fluent isiZulu and English speaker. Data were sorted and quality-checked by the KwaZulu-Natal project co-investigator, who is also a fluent isiZulu speaker. The data were analysed through thematic analysis (Braun & Clarke 2006).

This study was conducted with ethical consent from the University of South Africa, College of Education Ethics Committee (ref. number: 2014 July/90076087/MC).

## Findings from the field

This section presents findings of the study, based on the views of elders, on how people with disabilities were treated in the traditional communities. Verbatim quotes are presented in order to demonstrate the authenticity of data and express the elders’ views in their own words. However, it must be noted that the verbatim quotes have been translated from the isiZulu language (the language spoken by the elders) into English.

### Exclusion at birth

Among the nine elders, three revealed that children who were different from the norm would be killed at birth. The reasons that were given for this act were that these children were a curse from God. It was also believed that parents gave birth to a child with a disability because the ancestors were punishing them for wrongdoing. Victims of this exclusion included those who had a light complexion (people with albinism), those with any facial defects such as a cleft lip, those who had extra limbs or were missing limbs as well as those who demonstrated any sign of abnormality variance from the societal norm within a community.

‘When an unusual child was born, they would be killed without the mother knowing. This was done because it was believed that the disability was a curse from God and punishment from ancestors for a wrong committed by the parents.’ (Interviewee Gogo A, female, 85 years)

This elder further indicated that these forms of exclusion from the community were carried out in order to safeguard the community from calamities such as drought, earthquake and floods. Those who performed the killings were trusted to have a better understanding of what was best for the community. When asked if these practices were part of ubuntu, she said:

‘The way the disabled child was dealt with was inhumane but it used to happen.’ (Interviewee Gogo A, female, 85 years)

The response indicates hostility towards the powerless but the elder did not answer directly as to whether the act of killing was congruent with ubuntu or not.

In another community, elders revealed that if a disabled child had been born the parents were informed. The onus was then upon the parents to decide whether to keep the child or not.

Gogo B narrated:

‘It depended on the parent, the mother of the child. When God gave you a disabled child (*isidalwa*) you were to care for it forever and not go around complaining, you will stay with that child as a normal child. If God decided to take the child then it is God’s will.’ (Gogo B, female, 79 years)

There were cases of conflict regarding the parents’ decision to keep the disabled child. In some cases the mother who had decided to keep and care for her or him was occasionally abandoned by the husband and his family. Where a child was accepted in the family, his or her treatment was in some instances subhuman. The child was discriminated against and isolated from family and community functions. Gogo C explained that:

‘These people with disabilities who could not do anything for themselves and could not talk, in the past they used to be hidden in the house from the public’s sight. If there were an event or function at home they would be locked alone in one of the rooms until the function or event was over; that’s when she or he would be released from the room. Some were not even bathing; they were in trouble. You would find these disabled children … stinking with dirty clothing and *begxaza amathe* [drooling uncontrollably]. No one cared about them, not even the mother.’ (Gogo C, female, 95 years)

Interviewee Gogo D revealed in addition that:

‘The parents of the disabled child and their child were isolated or excluded from community activities.’ (Gogo D, female, 67 years)

When the researchers asked if this kind of treatment was portraying ubuntu, Gogo C said:

‘It was not Ubuntu my child, because these people were locked in the house for their whole life. The neighbours knew that there was a child who was not well in that house but they kept quiet; they kept quiet and never said anything. Maybe there is a celebration or a wedding by the neighbour and it is known that this child is abused; she gets locked in the house and given water, saying, here is some food, eat while we are away and when you sleep, sleep on the floor. The child would be woken up when the family gets back later on.’ (Gogo C, female, 95 years)

This statement reveals that children with disabilities were considered as sick people. The phrase ‘not well’ is usually used when someone is ill and needs medical attention. Interestingly, although children with disabilities were isolated and discriminated against in communities, the elders confirmed that the exclusion of people with disabilities was not ubuntu.

### Disabled people as unfit for marriage

As in most African cultures, the Zulu people observe traditional rituals and life events that people at certain developmental stages are required to undergo. One of these life events is marriage. Contrary to modern times where the family exerts less influence over whom their child should marry, in the past family members played a role in identifying a future spouse for their child. The process of spouse identification began while the girl was still young. She would be seen carrying out house chores, going to school, going to fetch water from the well and gathering firewood from the forest; then it would be decided that her skills would make her a valuable addition to the in-laws’ household.

People with disabilities did not benefit from this process of identification and matchmaking because they were hidden away from the community. Even those who were not concealed were not normally viewed as suitable partners because of ‘othering’. Powell and Menendian (2016:17) define *othering* as:

a term that not only encompasses the many expressions of prejudice on the basis of group identities, but … it provides a clarifying frame that reveals a set of common processes and conditions that propagate group-based inequality and marginality.

Gogo E revealed that people with disabilities did not move around much within their communities. Gogo E stated:

‘No, she or he would not go to church … because she or he would not be able to.’ (Gogo E, female, 69 years)

Gogo D related:

‘No, who will take you to church? The pastor will come home and give you Holy Communion if it is time for Holy Communion. These people with disabilities who cannot do anything for themselves and cannot talk, in the past they used to be hidden in the house from the public’s sight. … Some were not even bathing; they were neglected.’ (Gogo D, female, 67 years)

In terms of the preceding statements, it is evident that people with disabilities were excluded from social and religious activities because of their disabilities. This exclusionary practice is the reason why a group of Zulu people from four communities within KwaZulu-Natal interviewed were not familiar with how people with disabilities lived; this lack of knowledge could also be the cause for further alienation. When Gogo C was asked if she thought ubuntu values supported the marrying of people with disabilities, she responded by saying:

‘Oh no, if she can’t see what is happening around her, because you can’t be blind and marry. You can’t be married and not be able to see and serve your husband.’ (Gogo C, female, 67 years)

Mkhulu C revealed his belief that people with disabilities are not well; he stated:

‘*Hayi* [no], I can’t get married to a disabled girl because my family won’t accept her because she is not well.’

‘The neighbours knew that there was a child who was not well in that house but they kept quiet; they kept quiet and never said anything.’ (Mkhulu C, male, 69 years)

The researcher found it interesting that although she had clearly indicated that the enquiry was about how ubuntu values were applied, regardless of Gogo D’s supposed ubuntu values, the latter referred to the Bible to demonstrate why the community found it unacceptable for a person with a disability to marry. Gogo D narrated:

‘My daughter, in Genesis 2:18 the Bible says God gave Adam a helper because he saw him helpless so he was given a helper like you [*implying that the helper was supposed to be a woman*]. Then God said, I will make you a helper like you. He didn’t do it any other way; he took the woman and made her the man’s helper, and he didn’t say that the man would be the woman’s helper. So how is a disabled woman going to help her husband?’ (Gogo D, female, 67 years)

On the other hand, Mkhulu A expressed a totally different reason as to why people with disabilities were not regarded as appropriate marriage partners. He declared:

‘You see you have paid *lobola* [bride price] for a woman to be your third hand; secondly, you have parents that want to rest from doing things for you because your mother cannot keep waking up every morning and telling you to wake up. Your parents can no longer make food for you, wash and iron your clothes. The bride should help my parents. Yes, I can understand if disability came from an accident or injury and when you were already married to me.’ (Mkhulu A, male, 98 years)

People with disabilities were also regarded as not fit for marriage because they were considered as inactive and statues like furniture. As a result, such people were compared to furniture that just stays behind where the owner leaves it. Mkhulu B said:

‘I can’t bring furniture or a statue home, how will she be able to perform the bride’s chores? When we pay *lobola* we expect a wife who will perform her wifely duties.’ (Mkhulu B, male, 77 years)

However, in another community, it transpired that the ways the people with disabilities were treated regarding marriage depended on the nature of the disability. Gogo F responded:

‘Yes, they will grow and get married, depending on the disability. Most of them never got married; they stayed with their parents, except maybe if the disability were not severe, maybe just a knee or one eye affected. It depended on the disability.’ (Gogo F, female, 90 years)

### Inclusion of people with disabilities

While some lamented the exclusion of people with disabilities, others argued that there is inclusion of such people in their communities. The inclusion of a child with a disability depended on the mother’s love, support and choice. As Gogo C responded:

‘It depended on the love of the mother who gave birth to him or her … all this was a personal thing of the mother who gave birth to the child and the aunt who may have fallen in love with the child, who feels she must assist the mother in caregiving for the child; especially when she has to travel, the mother will ask her to assist in taking care of the child because she is travelling. Because some of such children were not able to feed themselves, fetching water and so on, they depended on a caregiver.’ (Gogo C, female, 95)

This shows that the inclusion of people with disabilities was not a communal responsibility in the way Gogo C puts it, but it was a family responsibility. Furthermore, this responsibility was carried by women. In initiation to puberty, girls with disabilities would be included, but there would be some tasks that they could not do because of the nature of their disability. As a result, they would be assisted by other girls.

Gogo B pointed out:

‘if you started your menstrual cycle (*ukuthomba*) you were supposed to sleep in one common place (*ukugonqa*) as girls of the same age. If you needed to go and fetch firewood (*ukuyotheza*), the girl with a disability could not participate because of her disability but she was taught (*ukuyalwa*) that because of what is happening now, this meant that she could fall pregnant and give birth to a child if she has sex. However, when they went to fetch firewood, it was said that because she was not able to fetch firewood (*ukutheza*) then other girls would do it on her behalf.’ (Gogo B, female, 79 years)

### Dehumanising labels

Social constructions and perceptions of people with disabilities are crucial as they involve the hidden negative emotions. Social perceptions are formed through the manner in which the society interacts with people with disabilities (Yeo & Moore 2003). Grotevant (2000) asserts that social perception and social activities determine the way in which people construct their identity. In the case of how a group of Zulu people from four communities within KwaZulu-Natal treated people with disabilities, research revealed negative results. Morris (1993:103) postulates that the negative treatment of people with disabilities is a result of the uneasy feelings that disability provokes among non-disabled people. In a study conducted at one university in South Africa, Kasiram and Subrayen (2013) highlighted how students with disabilities at the university were labelled as fools who were incapable beings. This demonstrates that these negative perceptions are not exclusive to non-educated villagers or elders from an archaic era but are socially embedded.

#### Unproductive and useless beings

Traditional communities relied on agricultural means of production prior to the advent of industrial and technological interventions. As far as some people with disabilities did not have the necessary physical strength that was essential for agricultural work, they were unable to contribute to food production (Kreitmair 2000). In the case of Germany, Dr Hinsen, an asylum director, advanced the same argument at a 1936 conference. He claimed that people with disabilities were ‘useless eaters’ who contributed nothing to the forms of production at that time. He further argued that the people with disabilities were a ‘burdensome existence’ (Kreitmair 2000:32). This perception could be elevated in societies in crises such as war or poverty; latent prejudices about disability and people with disabilities are likely to become explicit.

Mkhulu C stated:

‘These people cannot do anything for themselves. They are unproductive and useless people. You have to do all the work, bathe them, wash for them, dress them, feed them, phew!’ (Mkhulu C, male, 69 years)

#### Statues and furniture

Traditional communities have historically been obliged to do manual work in order to survive; the men had to go and hunt and the women were required to do agricultural work (Marks 1967). Although Mkhulu B had indicated that his communities regarded people with disabilities as fellow humans that deserve to be respected and treated the same as other community members, when asked if he would allow his son to marry a woman with a disability he made the following statement:

‘I can’t bring furniture or a statue home, how will she be able to perform the bride’s chores? When we pay *lobola* we expect a wife who will perform her wifely duties.’ (Mkhulu B, male, 77 years)

#### Banempene (*implying oversexualisation*)

Research results revealed that most families left their disabled family members alone at home. Others admitted to asking their neighbours to assist by taking care of their family member with a disability. In doing this, they exposed their female children to all kinds of abuse including rape and incest. Because people with disabilities were vulnerable and powerless when they reported the sexual abuse, the accused person would at times claim that she seduced him. Depending on the level of influence and relationship between the accused and the family of the person with the disability, the victim would be treated as the bad one who ‘asked’ for it (Theron & Phasha 2015). Nolte-Schamm (2006) narrates an experience of one sexual abuse victim who, upon reporting that her close relatives were abusing her, was told that she was lying and was labelled a ‘bitch’.

Gogo B related how one disabled girl was murdered by a mob because:

‘She was considered to be having *impene* [a word used when one has high libido perceived to be leading to promiscuity]. That girl accused respectable men in the community of raping her, so people were very angry with her and she was found in the thick bushes stoned to death. Nobody was arrested for her murder but some people were heard saying that she deserved to die because she seduced men.’ (Gogo B, female, 79 years)

These narrations reveal the vulnerability of people with disabilities in the hands of others who are supposed to care for and protect them. The derogatory terms being used when referring to them demonstrate the level at which communities perceive them negatively, regardless of asserting themselves as people with ubuntu values.

## Discussion and recommendations

The Zulu elders from four communities within KwaZulu-Natal reported that children with disabilities were killed, though there was no concrete moral value underpinning the action. The parents who had chosen to let their children live, while isolating them from the community activities, further denied the community a chance to gain a first-hand experience of how a person with a disability lives.

From the way the elderly spoke about disability, one can identify certain gender biases. It seems that female children were the ones who were mainly referred to when the exclusion of people with disabilities was discussed. In my view this constitutes violence against girls. Research indicates that women with disabilities and girls with disabilities experience violence and exclusion more than their male counterparts do (United Nations Convention on the Rights of People with Disabilities 2015). In addition, cultural initiation into adolescence and adulthood among children with disabilities was explained in reference to the disabled girl child while nothing was said about the disabled boy child. Seemingly, gender played a role in the exclusion or inclusion of a child with a disability. Considering women with disabilities as unmarriageable enforces the gender stereotypes where unremunerated, and undervalued, productive and reproductive roles performed by women are a prerequisite for marriage.

Meanwhile, Ngubane-Mokiwa (2013) reveals that the negative perception and treatment of people with disabilities is not always because of cruelty but rather points to fear of the unknown. Some people have not had close contact with people with disabilities so they do not understand them and how they live. According to Kreitmair (2000:18) in his work *In Fear of the Frail*, Adolf Hitler of Germany viewed people with disabilities as a health threat because of the ‘evils of incurable illness and disability’. Hitler asserted that taking time to care for people with disabilities deprived the healthy ones of enough time to be productive for the benefit of the society and the country.

The analysis of the findings highlights a disturbing confusion as regards ubuntu among the Zulu communities researched. Though it is not clear from the elders’ responses as to how they felt about the exclusion of people with disabilities during those years, it was evident that their current perspectives were informed by modern values and beliefs about what it means to be humane. They may have performed inhumane practices on those with disabilities because their context at that time allowed them to. Consequently it is obvious that understanding and application of ubuntu changes with context. At the end of the study, one issue became clear: that there is a need to work with communities to renegotiate the meaning and application of ubuntu. There is also a need to restructure how societies respond to people with disabilities to achieve full inclusion.

## Conclusion

This study has explored the inclusion and exclusion of people with disabilities among a group of Zulu people from four communities within KwaZulu-Natal. The findings indicated that people with disabilities were excluded from and by their communities. Their exclusion was because of their parents’ powerlessness in the face of tradition. This research then offers empirical evidence as well as a theoretical explanation for the process of their exclusion. Findings also indicate that the exclusion of people with disabilities was a misrepresentation and misinterpretation of ubuntu. Exclusion from society is oppression and it dehumanises those excluded. There is a need for traditional communities to conduct ongoing conversations on how vulnerable people can be guaranteed their human rights.
